# Neurological soft signs are increased in migraine without aura: relationship with the affective status

**DOI:** 10.1007/s10072-022-06143-3

**Published:** 2022-05-18

**Authors:** Lucio Tremolizzo, Daniele Selvatico, Federico Emanuele Pozzi, Diletta Cereda, Jacopo Cosimo DiFrancesco, Lorenzo Fumagalli, Carlo Ferrarese, Ildebrando Appollonio

**Affiliations:** 1 Neurology Unit, ASST Monza, San Gerardo Hospital, Monza, Italy; 2 School of Medicine and Surgery, University of Milano-Bicocca, Monza, Italy; 3 Milan Center for Neuroscience (NeuroMI), Milan, Italy

**Keywords:** Neurological soft signs, Migraine, Headache, Brain imaging, Depression, Anxiety

## Abstract

**Introduction:**

Neurological soft signs (NSS) are subtle non-localizing sensorimotor abnormalities initially reported as increased in primary headache patients. The aims of this study were confirming with full power NSS increased expression in migraine and, collaterally, determining if psychiatric traits or white matter lesions at brain imaging could influence this result.

**Methods:**

Forty drug-free episodic migraine outpatients (MH) were recruited with 40 matched controls. NSS were determined by the 16-item Heidelberg scale; depression, anxiety and QoL by the HAM-D; the STAI-X1/X2; and the SF36, respectively. The Fazekas scale on brain MR studies was applied in *n* = 32 MH, unravelling deep white matter signal alterations (DWM). MH characteristics, including the headache disability inventory (HDI), were recorded.

**Results:**

NSS were 46% increased in MH vs. controls (*p* = 0.0001). HAM-D and STAI-X1/X2 were increased in MH, while SF36 was unchanged, but they all failed to influence NSS, just as MH characteristics. NSS scores were increased in MH-DWM + (*n* = 11, + 85%) vs. MH-DWM − (*n* = 21, + 27%) vs. controls (*p* < 0.0001). NSS increased expression in MH was influenced by DWM, while psychiatric traits and headache characteristics failed to do so.

**Discussion/conclusions:**

NSS are increased in MH and probably not influenced by the affective status, possibly marking a dysfunction within the cerebellar-thalamic-prefrontal circuit that may deserve further attention from the prognostic point of view.

## Introduction

Neurological soft signs (NSS) are subtle non-localizing sensorimotor anomalies whose clinical and biological significance is still a matter of discussion [1]. NSS assessment encompasses different domains, including motor coordination and sensory integration, but neurologists do not usually assess NSS during the routine neurological examination or at least do not apply one of the many different evaluation scales that have been developed to quantify NSS expression [2]. Since the very beginning, in fact, the presence of a high level of NSS expression has always been considered as a prerogative of psychiatric disorders. In 1975, Tucker and colleagues used the term NSS for the first time, identifying, in a group of 109 first-episode psychotic patients, a dramatic increase of subtle neurological semeiotic anomalies with respect to a control group of non-psychotic people [3]. Subsequent studies documented increased expression of these signs also in people who had a familiarity for psychosis, despite not suffering from the disease themselves [4]. Furthermore, other studies underlined the increase of NSS in people with mild-moderate intensity of the psychotic symptoms and, in general, in people at risk of developing schizophrenia at any stage of their illness. Therefore, for all these reasons, NSS have been considered as possible psychotic endophenotypes. Nevertheless, since then, NSS have been studied in other psychiatric diseases ranging from obsessive–compulsive disorder [5], to borderline personality disorder [6], and ADHD [7], among others, globally showing — albeit with mixed results — increased expression; this eventually questioned NSS specificity and sustained the intriguing possibility that NSS might mark shared traits among different psychiatric diseases. Possibly, further enlightenment on these issues might come from the understanding of the plausible “mild localizatory” value of these non-localizatory signs [1]. In particular, studies on healthy subjects, where NSS can be measured, show that their expression can be related to a cerebellar contribution [8] and to white matter microstructure variations in subcortical and cortical sensorimotor regions [9].

We previously reported, for the first time in a pilot study, a significant increase of NSS in headache patients compared to headache-free subjects [10]. Notably, headache as a disease presents a significant comorbidity with psychiatric disorders that increases with the number of attacks [11, 12], with some authors arriving to propose it as a symptom of a badly adaptive stress managing attitude [13]. Even setting aside this issue, most cases of primary headache seen in neurological outpatient practice co-express at least mild anxiety or depressing symptoms that are commonly worth of mixed prophylactic drug approaches, such as amitriptyline [14]. We therefore hypothesized that our previous results concerning NSS in headache patients could be eventually influenced by the co-present psychiatric dimensional variables. This hypothesis was also strengthened by the fact that NSS followed the same increasing trend in both migraine (MH) and tension-type headache (TTH) without distinctions between two such different disorders [10]. Furthermore, in this preliminary report, NSS were also related to the presence of white matter hyperintensities (WMH), in particular to deep white matter (DWM) signal alterations [10], in line with what was previously more precisely demonstrated [9].

Based on our pilot data, we calculated the power for a confirmation study, focusing on MH patients, since the presence of brain micro-structural anomalies [15] and the existence of genetic traits have received more attention in the literature in this headache type [16]. The aims of the present study were the following: (1) confirming the increase of NSS in MH patients versus matched headache-free controls, by including only prophylactic drug-free patients; (2) verifying the impact of anxiety and depression on NSS expression; (3) unveiling possible relationships between NSS and WMH by retrospectively examining brain MR performed by these patients.

## Materials and methods

### Recruited patients and brain imaging

Following ethical committee approval (Monza e Brianza, Italy; protocol MIGRASoft) and informed consent, 40 consecutive patients affected by episodic migraine without aura (MH) were recruited during the headache-free period (i.e., they were headache-free at least since the day before the assessment) from the outpatients afferent to the Neurology Unit of the San Gerardo Hospital (Monza, Italy). Inclusion criteria were the following: (a) diagnosis of migraine (MH) without aura according to current criteria from the International Headache Society (https://www.ichd-3.org/); (b) age > 18 and < 75 years old; (c) number of attacks between 1 and 14 per month; (d) no current prophylaxis or other psychoactive medications; (e) no previous history of neurological or psychiatric disorders other than headache; (f) a fully normal standard neurological examination. The average number of attacks/month and the number of over-the-counter medication/month, as a measure of headache severity, referring to the previous three months period were recorded. Data regarding duration of headache was not considered relevant and thus not included in the analysis. Patients were also asked to complete the Headache Disability Inventory (HDI) [17].

Healthy controls (CTRL, *n* = 40) were rigorously age- (± 2 years), education- (± 3 years) and sex-matched, and they were recruited from the spouses of other neurological outpatients. They had no previous history of neurological or psychiatric disorder (including episodic frequent headache), nor were they under psychoactive medications. Demographic and clinical data are included in Table 1.

**Table 1:** Demographic and clinical variables of the recruited subjects. Data are shown as mean ± *SD* (range). CTRL, controls; HDI, headache disability inventory; MH, migraine patients; N/A, not applicable

**Variables**	**MH** (*n* = 40)	**CTRL** (*n* = 40)
Sex, F (%)	32 (80%)	32 (80%)
Age, yrs	37.6 ± 12.3 (20–74)	37.8 ± 12.3 (20–76)
Education, yrs	13.3 ± 3.4 (5–23)	14.5 ± 3.7 (5–22)
**# **attacks/month	7.7 ± 4.1 (1–14)	N/A
**# **acute headache treatment/month	7.5 ± 5.2 (0–30)	N/A
HDI	42.7 ± 15.8 (6–88)	N/A

A brain MR scan (1.5 T) was obtained in *n* = 32 MH patients (coverage 80%, obtained retrospectively but performed within 6 months from enrolment), including axial FLAIR sequence (slice thickness 5 mm with a gap of 1 mm; TR 6000 ms/TE 120 ms; field of view: AP 230 mm/RL 183 mm/FH155 mm) that provided whole brain coverage. Images were assessed blindly by two independent neurologists with experience in brain imaging analysis, and the Fazekas scale applied to qualitatively score white matter hyperintensities (WMHs), dividing them in periventricular white matter (PVWM) and deep white matter (DWM) signal alterations [18].

### NSS scales and comorbid traits

Besides the complete standard neurological examination, all patients were — subsequently — evaluated for NSS. The examination was carried out by a single neurologist after training, in a calm environment, without interruptions or additional observers. As in our pilot work [10], we choose the 16-item Heidelberg scale [19] since, unlike other batteries, it excludes primitive reflexes, more properly to be considered as markers of cognitive and upper motor neuron dysfunctions [20]. As so, they are qualitatively different from the core NSS, mainly addressing sensorimotor integration and coordination. As reported before, the five different subdomains of the scale were also analyzed separately: (1) motor coordination, (2) integrative function, (3) complex motor tasks, (4) right/left and spatial orientation, (5) hard signs. The scale was translated in Italian from the original German version by one of the authors with working proficiency in both languages, and its adherence to the original was assessed with back-translation.

Finally, all recruited subjects were asked to complete the Hamilton rating scale for depression (HAM-D) and the State-Trait Anxiety Inventory form X (STAI-X), calculating state anxiety (STAI-X1) and trait anxiety (STAI-X2). Furthermore, recruited subject completed the SF36 for quality of life (QoL) determination, allowing us to calculate the Physical Component Summary (SF36-PCS) and the Mental Component Summary (SF36-MCS). All questionnaires were completed by the subjects during the visit under the supervision of the visiting neurologist to clarify possible doubts.

### Power calculation and statistical analysis

Power for this study was calculated based on our previous results, including 20 MH patients and an equal number of matched controls [10]. Hypothesizing an increase of 50% of NSS scores in MH patients versus CTRL, alpha 5% and beta 10%, current sample size (*n* = 40 patients and *n* = 40 controls) was consistent with 100% power. Statistical analysis was performed by SPSS version 23. Data are reported as mean ± standard deviation. NSS differences among the recruited groups were assessed by ANCOVA followed by Bonferroni post hoc test, controlling for age and education, and clinical scores as covariates. Two-tailed Student’s *t*-test, ANOVA followed by Bonferroni and two-tailed Pearson analysis of correlation were used as appropriate.

## Results

**Fig. 1:**
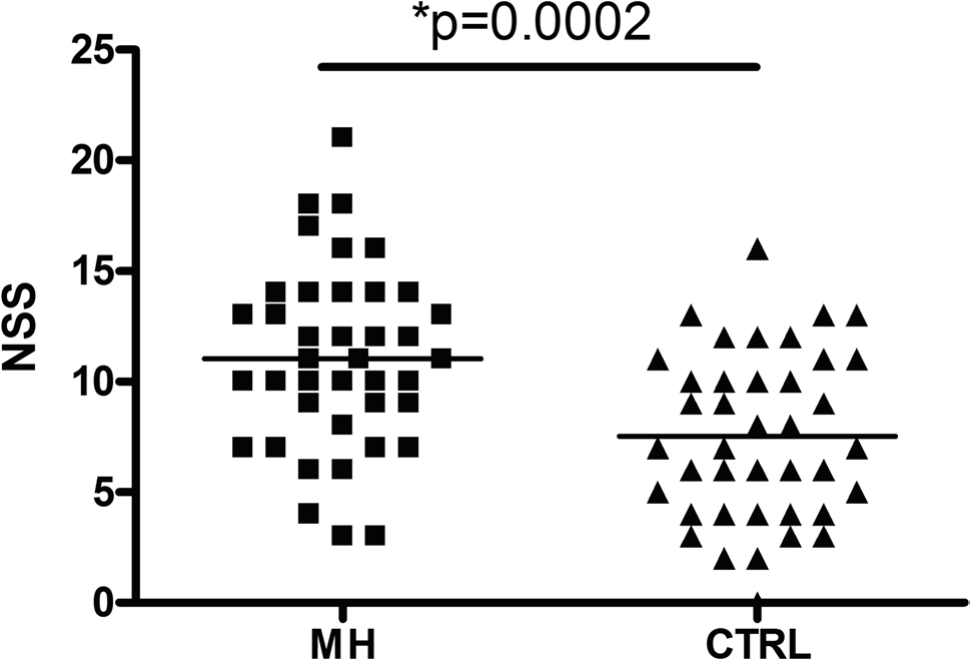
Distribution plot of neurological soft signs (NSS) scores in migraine patients (MH) versus controls (CTRL). *Two-tailed Student’s *t*-test (*t* = 3.959, *df* = 78)

NSS were 46% increased in MH versus CTRL (shown in Fig. 1; *p* = 0.0001). Analyzing each NSS subdomain separately, differences were found only for “integrative functions” (+ 97%, *p* = 0.0007) and “right/left and spatial orientation” (+ 104%, *p* = 0.0001), with a tendency toward a difference for “hard signs” (+ 37%, *p* = 0.064). No differences were found for “motor coordination” and “complex motor tasks.”

In both patients and controls, NSS showed a positive linear correlation with age (*r* = 0.52, *p* = 0.0005 and *r* = 0.61, *p* < 0.0001, respectively), while a negative correlation was present for education (*r* =  − 0.47, *p* = 0.002 and *r* =  − 0.46, *p* = 0.003, respectively). On the other hand, no correlations were present between NSS and HDI, number of headache attacks, and symptomatic drugs. The high number of symptomatic drugs taken in a month (see Table 1) raises the issue of a concomitant medication-overuse headache (MoH), which is indeed a form of chronic headache. Only one patient reported *n* = 30 drugs/month; eliminating this patient, the number of symptomatic drugs was 6.9 ± 3.6 (0–15), and only *n* = 7 MH patients reported from 10 to 15 drugs/month, including a variable number of both triptans and NSAIDs from month to month in each patient. Therefore, in the worst-case scenario, the number of patients potentially co-expressing MoH was *n* = 8 (20%). A separate assessment was made with the exclusion of these cases, but it did not alter the results of the subsequent analysis; therefore, we chose to present the findings obtained with the complete sample of patients.

**Fig. 2:**
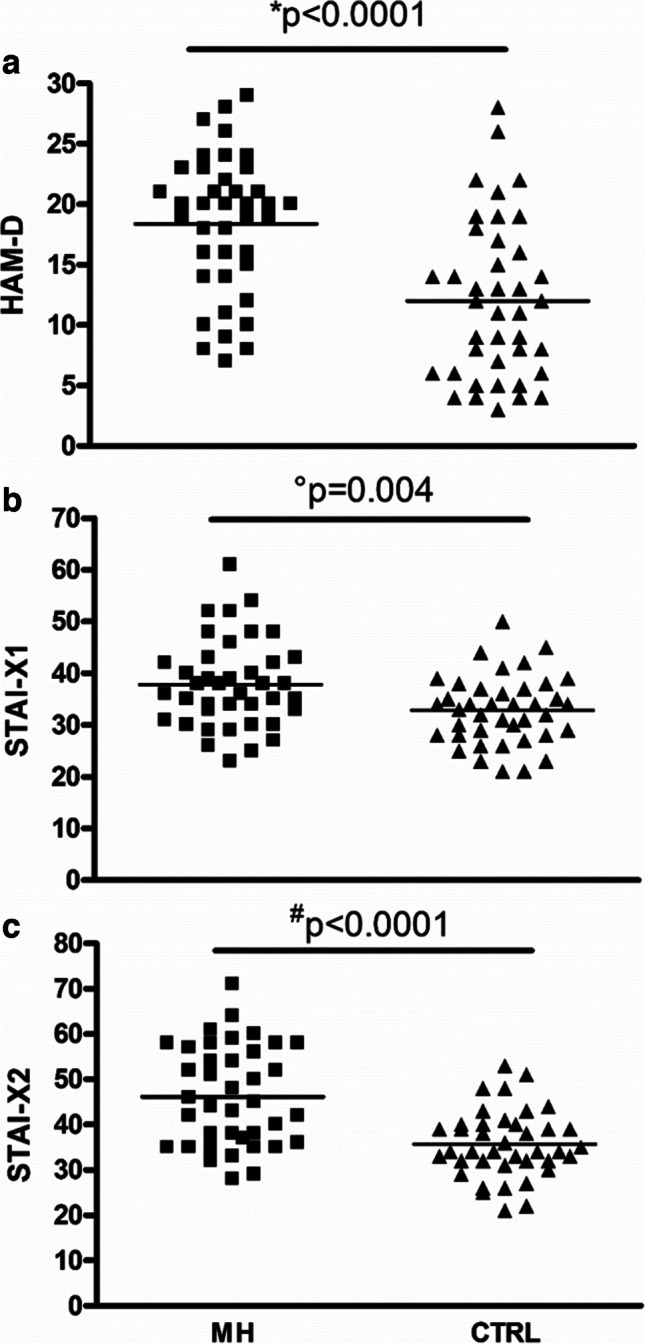
Distribution plot of (**a**) Hamilton Rating Scale for Depression (HAM-D), (**b**) State-Trait Anxiety Inventory form X for state anxiety (STAI-X1), and (**c**) State-Trait Anxiety Inventory form X for trait anxiety (STAI-X2) scores in migraine patients (MH) with respect to controls (CTRL). Two-tailed Student’s *t*-test: *t = 4.661, *df* = 78; °*t* = 2.951, *df* = 78; ^#^*t* = 4.953, *df* = 78

Mean HAM-D score was increased in MH vs. CTRL (+ 53%, *p* < 0.0001; shown in Fig. 2a), as was STAI-X1 (+ 15%, *p* = 0.004; shown in Fig. 2b) and STAI-X2 ones (+ 29%, *p* < 0.0001; shown in Fig. 2c). Conversely, no changes were shown for SF36-PCS and SF36-MCS scores in MH vs. CTRL (48 ± 15.9 vs. 51.4 ± 6.6, *p* = 0.21 and 52.6 ± 16.7 vs. 49.9 ± 8.7, *p* = 0.35, respectively). Interestingly, a mild correlation emerged only for MH between NSS and HAM-D scores (*r* = 0.34, *p* = 0.03). Finally, the significant increase of NSS in MH vs. CTRL was confirmed even after controlling for HAM-D, STAI-X1, STAI-X2, SF36-PCS, SF-36-MCS, age, and education (*p* = 0.005).

**Fig. 3:**
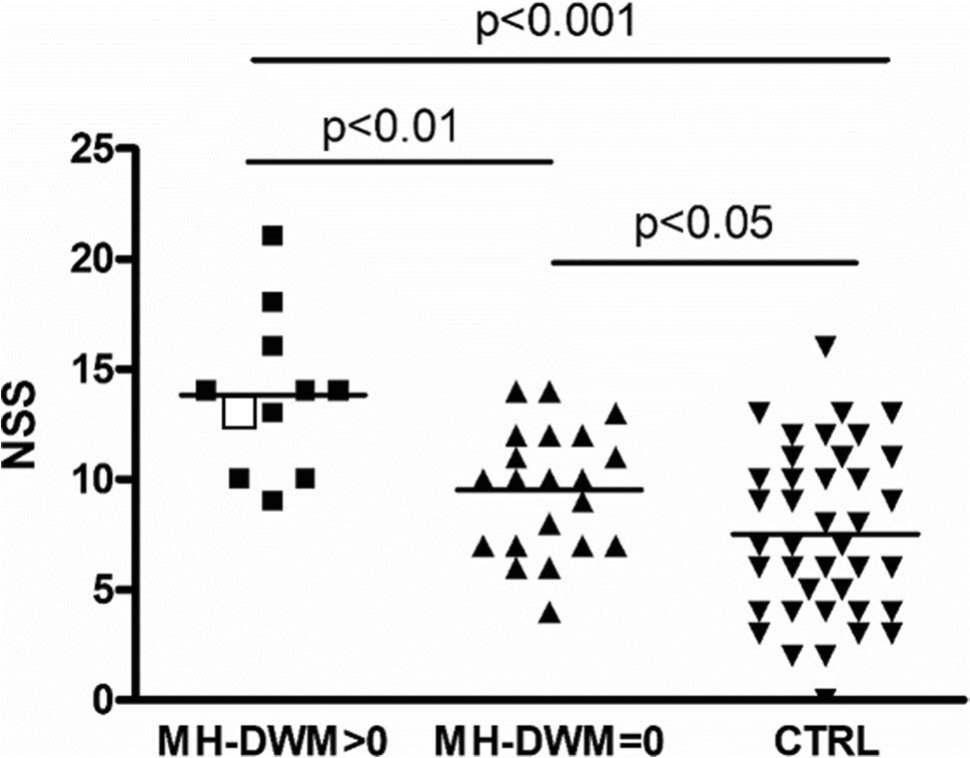
Neurological soft signs (NSS) are increased in migraine patients with deep white matter signal alterations (MH-DWM > 0) vs. migraine patients without such alterations (MH-DWM = 0) vs. controls (CTRL). *p* < 0.0001 at ANOVA, followed by Newman-Keuls multiple comparison test (*p* value reported). The white square indicates one MH-DWM > 0 patient with DWM score = 2 (all the other patients had a DWM score = 1)

WMHs were found in 11 out of 32 MH patients (34%). At the Fazekas score, none of the patients displayed PVWM signal alterations. Almost all positive patients displayed a DWM signal alteration score of 1, while only one patient had a score of 2 (white square in Fig. 3; none had a score of 3). NSS scores were increased in MH-DWM + (+ 85%) vs. MH-DWM − (+ 27%) vs. CTRL (*p* < 0.0001 at ANOVA, shown in Fig. 3). Three MH patients were taking medication for hypertension, but no relationship with DWM was noted. One MH patient was taking both medication for hypertension and diabetes, but brain imaging was not available.

## Discussion

In this study, we provide further evidence for increased NSS in migraineurs, which was the main aim of our work. We included prophylactic drug-free patients with episodic migraine without aura, during the headache-free period, calculating sample size based on our previous preliminary report [10]. In this quite rigorous setting, NSS scores were increased, albeit the important overlap between the two groups plausibly limits any direct attempt of clinical application. NSS also did not correlate with MH clinical variables, including headache-related disability (HDI). Potential MoH presence in our setting failed to drive NSS expression, but a limitation of this study consists in not having rigorously recorded MoH presence, which was hypothesized merely on a self-reported questionnaire, estimating the number of symptomatic drugs taken during the previous three months; however, since the exclusion of these patients did not alter the results and the MoH co-diagnosis was not certain, we chose to present the data obtained in the whole sample. Future studies should use more rigorous tools, such as the headache diary, incorporating other important data, such as headache intensity, which was not included in this study. The same limit applies to the number of migraine attacks that was reported — on average — based on patient’s memory. On the other hand, age and education — as previously reported — were NSS determinants and were necessarily included in the covariate analysis [10]; therefore, no other correlation of age with clinical and radiological characteristics was performed. The specificity of this finding has still to be determined, and for this reason, we are currently collecting NSS data on episodic frequent tension-type headache patients.

In any case, aims of this study were also to verify the impact on NSS in MH patients of the following: (a) anxiety and depression and of (b) diffuse white matter signal alterations; both (a) and (b) are, in fact, often present in comorbidity to MH and might influence mild sensorimotor anomalies. Regarding the psychiatric traits, our MH patients expressed significantly higher score in anxiety and depression scales compared to controls, but both parameters failed to modify NSS increase in the covariate analysis. This is somehow in line with the literature, where, overall, less inconsistency is found when considering disorders that dictate profound brain circuit modifications, such as schizophrenia. Nevertheless, one limit of this study is represented by the fact that the presence of a psychiatric comorbidity cannot be evaluated just by means of a simple scale; this is particularly true when considering that self-administered tools could be over- or underestimating the severity of a clinical trait or disease [21]. The fact that HAM-D scores mildly correlated with NSS ones could, perhaps, be a reason for looking deeper into this matter before concluding. In any case, neither headache associated disability, nor QoL, two independent mood-related concepts were associated to NSS expression.

Although the relationship between WMH and headache is still under debate, with some authors reporting an increase in MH patients compared to controls [22], these alterations seemed to show a clear influence on NSS expression in our study; unfortunately, the impossibility of obtaining imaging in healthy controls did not allow to include their data in covariate analysis. Nevertheless, NSS was increased in MH patients with WMH compared to controls: Most notably, NSS was also increased in MH patients with WMH vs. migraine patients without such alterations. However, apparently against this conclusion is the fact that we report a mild but significant NSS increase also in WMH-free patients with respect to controls: Given the exploratory value of our data, we could hypothesize that high-field MR studies might be able to decrease progressively the number of WMH-free patients.

This result raises an immediate question about the real localizing value of this non-localizatory chapter of neurological semeiotics. In fact, NSS have been theorized in the field of psychiatry with the intent of creating a potential bridge between the two disciplines: however, the fact that NSS have been overtly developed without embracing a “localizing” perspective has probably always kept neurologists far from bestowing value on them. We decided anyway to adopt this approach considering that (a) some NSS gestures are clearly shared with the “hard” neurological semeiotics (e.g., finger-to-nose, or the Luria fist-edge-palm test), and (b) the standard neurological examination is non-informative, by definition, in primary headache disorders. Applying NSS semeiotics in this setting, we conclude that it is very similar to the standard neurological one, with the differences of a poor localizatory value, strongly increased “sensitivity” applied by the rater during the scoring process, and a semi-quantitative approach. Some authors already hypothesized that NSS mark an increasing dysfunction within the cerebello-thalamo-prefrontal circuit [1], the disruption of which produces “cognitive dysmetria,” i.e., the difficulty in prioritizing, processing, coordinating, and responding to information that has been hypothesized to be the central cognitive deficit characterizing schizophrenia as a brain disorder [23]. This “poor mental coordination” is distinct from the executive functions, since it involves several mental functions, such as memory, attention, emotion, and motor activity, and several neural networks, such as not only prefrontal cortex, but also subcortical nodes as well [23]. Thus, the NSS increase we found in MH patients might be marking a vulnerability along this circuit, albeit the results of a recent study argue against the presence of mild cerebellar dysfunction in these patients [24]; our data regarding the distribution of WMH, with all the limitations already described, should be considered as preliminary and therefore do not allow to test this hypothesis. Perhaps, further studies on NSS in different headache types will be helpful for settling this issue.

One important limit that questions the possibility of a routine clinical use of NSS scales consists in the relatively long duration when considering the relatively short time available in any outpatient clinical setting. Even so, based on these premises on NSS role, we recently assessed NSS in patients affected by different types of dementia, characterized by different cortical or subcortical involvements. After correcting for Mini-Mental State Examination (MMSE), Frontal Assessment Battery (FAB), Neuropsychiatric Inventory-10 items (NPI-10), disease duration, and age, we found a clear difference in NSS expression between patients affected by Alzheimer’s disease, frontotemporal dementia, and corticobasal syndrome that certainly deserves further attention [25].

A final issue which remains unanswered consists in determining if NSS may play a role in predicting MH response to prophylactic treatment. In fact, if NSS mark the presence of more pronounced brain circuit alterations, we may hypothesize that these patients could have different prognoses and responses to treatments, considering that responders undergo to specific brain changes with respect to non-responders [26]. Analogously, the possibility that the NSS expression itself could change over time, perhaps contextually with changes in disease activity or therapeutic interventions, exists and might be investigated [27]. All these questions, eventually regarding the real relevance of NSS in primary headaches [28], will be answered by future prospective studies, perhaps incorporating also genetic determinants, such as the ApoE genotype [29].

## Conclusions

In our study, we confirmed that NSS are increased in episodic MH patients without aura; however, their expression does not seem to be driven by comorbid psychiatric traits such as anxiety and depression. Our data showed that NSS might be also increased in MH patients with deep WMH compared to MH patients without these alterations, consistent with the hypothesis that NSS may mark a dysfunction within the cerebellar-thalamic-prefrontal circuit. However, further studies are needed to elucidate the prognostic role of NSS in migraine and their relationship with subcortical neuroimaging abnormalities.

## Data Availability

The data that support the findings of this study and the Italian translation of the Heidelberg NSS Scale are available from the corresponding author upon reasonable request.
